# Identification of (2*S*,3*S*)-β-Methyltryptophan as the Real Biosynthetic Intermediate of Antitumor Agent Streptonigrin

**DOI:** 10.1038/srep20273

**Published:** 2016-02-05

**Authors:** Dekun Kong, Yi Zou, Zhang Zhang, Fei Xu, Nelson L. Brock, Liping Zhang, Zixin Deng, Shuangjun Lin

**Affiliations:** 1 State Key Laboratory of Microbial Metabolism, Joint International Laboratory on Metabolic & Developmental Sciences, School of Life Sciences & Biotechnology, Shanghai Jiao Tong University, 800 Dongchuan Road, Shanghai 200240, P. R. China; 2 College of Life Science, Hebei University, Baoding 071002, P. R. China

## Abstract

Streptonigrin is a potent antitumor antibiotic, active against a wide range of mammalian tumor cells. It was reported that its biosynthesis relies on (2*S*,3*R*)-β-methyltryptophan as an intermediate. In this study, the biosynthesis of (2*S*,3*R*)-β-methyltryptophan and its isomer (2*S*,3*S*)-β-methyltryptophan by enzymes from the streptonigrin biosynthetic pathway is demonstrated. StnR is a pyridoxal 5′-phosphate (PLP)-dependent aminotransferase that catalyzes a transamination between L-tryptophan and β-methyl indolepyruvate. StnQ1 is an *S*-adenosylmethionine (SAM)-dependent *C*-methyltransferase and catalyzes β-methylation of indolepyruvate to generate (*R*)-β-methyl indolepyruvate. Although StnR exhibited a significant preference for (*S*)-β-methyl indolepyruvate over the (*R*)-epimer, StnQ1 and StnR together catalyze (2*S*,3*R*)-β-methyltryptophan formation from L-tryptophan. StnK3 is a cupin superfamily protein responsible for conversion of (*R*)-β-methyl indolepyruvate to its (*S*)-epimer and enables (2*S*,3*S*)-β-methyltryptophan biosynthesis from L-tryptophan when combined with StnQ1 and StnR. Most importantly, (2*S*,3*S*)-β-methyltryptophan was established as the biosynthetic intermediate of the streptonigrin pathway by feeding experiments with a knockout mutant, contradicting the previous proposal that stated (2*S*,3*R*)-β-methyltryptophan as the intermediate. These data set the stage for the complete elucidation of the streptonigrin biosynthetic pathway, which would unlock the potential of creating new streptonigrin analogues by genetic manipulation of the biosynthetic machinery.

Streptonigrin (STN, **1**) is a highly functionalized aminoquinone alkaloid produced by *Streptomyces flocculus*^1^,^2^. Structurally and biogenetically, STN, lavendamycin (**2**), streptonigron (**3**), and related congeners constitute the “streptonigrinoids”-family of natural products (Fig. 1)^3^,^4^,^5^,^6^. STN is composed of four six-membered rings, among which the aminoquinoline and fully substituted pyridine rings are almost co-planar, while the multi-substituted phenyl ring faces them perpendicularly^7^,^8^.

STN has shown antiviral activity, potent and broad spectrum activities against bacteria and fungi^9^,^10^ as well as potent activity against a wide range of tumors^9^,^10^,^11^,^12^,^13^. Above all else, the antitumor mechanisms of STN have been extensively investigated^14^. STN induces DNA single- and double-strand breaks, unscheduled DNA synthesis, and DNA complex formation^15^. Moreover, it causes sister-chromatid exchanges and chromosomal aberrations and it can inhibit the topoisomerase II and block the synthesis of DNA and RNA^16^,^17^,^18^.

Several total syntheses of STN were successfully conducted allowing for the possibility of structural modifications with the objective to obtain new bioactive streptonigrinoids^19^,^20^,^21^,^22^. In quest of new bioactive streptonigrinoids, the biosynthetic pathway of STN has been studied by detailed feeding experiments with isotopically labeled precursors^23^,^24^,^25^,^26^,^27^,^28^,^29^,^30^. Initial biosynthetic studies of STN in *S. flocculus* suggested that the 4-phenyl picolinic acid moiety is derived from l-tryptophan via β-methyltryptophan as an intermediate^26^. Subsequently, (2*S*,3*R*)-β-methyltryptophan (MeTrp) was obtained from l-tryptophan (l-Trp), mediated by cell-free extracts of the STN-producing strain *S. flocculus* ATCC 13257^31^. Taken together with the identification of (2*S*,3*R*)-β-MeTrp in STN fermentation cultures^28^,^29^, (2*S*,3*R*)-β-MeTrp has been regarded as the biosynthetic precursor of STN^31^,^32^. In this study, the biosynthesis of (2*S*,3*R*)- and (2*S*,3*S*)-β-MeTrp by enzymes from the biosynthetic pathway of STN was characterized *in vitro*. Furthermore, experimental evidence is presented that implicates (2*S*,3*S*)-β-MeTrp rather than (2*S*,3*R*)-β-MeTrp as the true biosynthetic intermediate of the STN biosynthetic pathway.

Recently, a biosynthetic gene cluster for the production of STN has been cloned and identified that consists of 48 genes^33^. Bioinformatic analysis of the *stn* gene cluster revealed a three-gene cassette (*stnQ1-stnK3-stnR*) encoding an *S*-adenosylmethionine (SAM)-dependent *C*-methyltransferase, a cupin-fold epimerase, and a pyridoxal 5′-phosphate (PLP)-dependent aminotransferase, respectively. This gene cassette is homologous to the *marI-marH-marG* cassette that generates (2*S*,3*S*)-β-MeTrp for the biosynthetic pathway of maremycins (Figure S1; identity/similarity: 64%/77% for StnQ1/MarI, 82%/86% for StnK3/MarH, and 64%/73% for StnR/MarG)^34^. The bioinformatic data suggests that the *stnQ1-stnK3-stnR* cassette is responsible for the biosynthesis of (2*S*,3*S*)-β-MeTrp as well.

## Results and Discussion

### Functional characterizations of StnR/Q1/K3

In order to characterize the enzymes of this cassette *in vitro*, the three genes were individually overexpressed in *E. coli* BL21(DE3) and the corresponding *N*-terminally His_6_-tagged proteins were purified to near homogeneity (Figure S2). Subsequently, the enzymatic activities of these three proteins were evaluated following a previously described procedure^34^. StnR catalyzed the transamination of l-Trp to generate indolepyruvate when it was incubated with the amino acceptor α-ketoglutarate (Fig. 2a, trace iv). StnQ1 catalyzed a methyl transfer from SAM onto the β-carbon of indolepyruvate to form β-methyl indolepyruvate (Fig. 2a, trace v), but it could not catalyze the direct methylation of l-Trp under the same conditions. When StnQ1 and StnR were incubated with l-Trp and SAM in a one-pot reaction, (2*S*,3*R*)-β-MeTrp was produced without the addition of an additional amino donor or acceptor (Fig. 2b, trace iv). This result indicates that StnQ1 catalyzes the methylation of indolepyruvate with (*R*)-specificity generating (*R*)-β-methyl indolepyruvate, and that StnR is able to catalyze the transamination of (*R*)-β-methyl indolepyruvate using l-Trp as an amino donor. The epimerase StnK3 can catalyze the inversion of (*R*)-β-methyl indolepyruvate to give rise to its (*S*)-isomer^34^ and accordingly (2*S*,3*S*)-β-MeTrp was obtained when l-Trp and SAM were incubated with StnK3, StnQ1 and StnR (Fig. 2b, trace v). All of these data are in accordance with those for the MarG/H/I system that have been reported previously^34^.

### Stereoselectivity of the aminotransferase StnR

The aminotransferase StnR specifically requires l-Trp as its amino donor substrate, but cannot accept d-Trp instead. Interestingly, each of the (2*S*)-β-MeTrp isomers could be obtained from l-Trp by using either two (for 3*R*-configuration) or all three (for 3*S*-configuration) of the enzymes from the StnR/Q1/K3 system. The major question remaining is, whether StnR exhibits an inherent stereoselectivity towards one of the corresponding β-methyl indolepyruvate epimers as amino acceptors. To answer this question, the rates of StnR-catalyzed transamination of (*R*)- and (*S*)-β-methyl indolepyruvate have to be compared. Unfortunately, enantiopure (*R*)- and (*S*)-β-methyl indolepyruvate are not available, because keto-enol tautomerism makes β-methyl indolepyruvate prone to racemization. Instead, the stereoselectivity of StnR was deduced by comparing the rates of (2*S*,3*R*)- and (2*S*,3*S*)-β-MeTrp formation from racemic β-methyl indolepyruvate under StnR catalysis. For this purpose, (*R*)-β-methyl indolepyruvate was synthesized by StnQ1-catalyzed methylation of indolepyruvate and racemized at room temperature. The generation of racemic β-methyl indolepyruvate was evidenced by the formation of almost equal amounts of (2*S*,3*R*)- and (2*S*,3*S*)-β-MeTrp in an assay using excess StnR (20 μM) which ensures high conversion of both β-methyl indolepyruvate isomers (Fig. 2c, trace iii). In follow-up experiments, the amount of StnR was reduced to 2 μM and the reactions were quenched at different time points (0.5, 1.0, 2.0, 4.0, and 8.0 h). It became apparent that (2*S*,3*S*)-β-MeTrp was predominantly produced in the early stages of the reaction (0.5-4 h, Fig. 2c, trace iv-vii) while (2*S*,3*R*)-β-MeTrp production ramped up afterwards. Exemplarily, (2*S*,3*S*)-β-MeTrp was generated as a sole product at 0.5 h incubation time with only trace amounts of (2*S*,3*R*)-β-MeTrp present (Fig. 2c, trace iv). Overall, these data clearly demonstrate that StnR is not only l-(*S*)-specific for the amino donor, but also an enantioselective transaminase regarding the stereochemistry of the β-substituent that prefers (*S*)-β-methyl indolepyruvate over its (*R*)-epimer as an amino acceptor.

### Inactivation of *stnQ1* in the STN-producing strain

**Figure 1:**
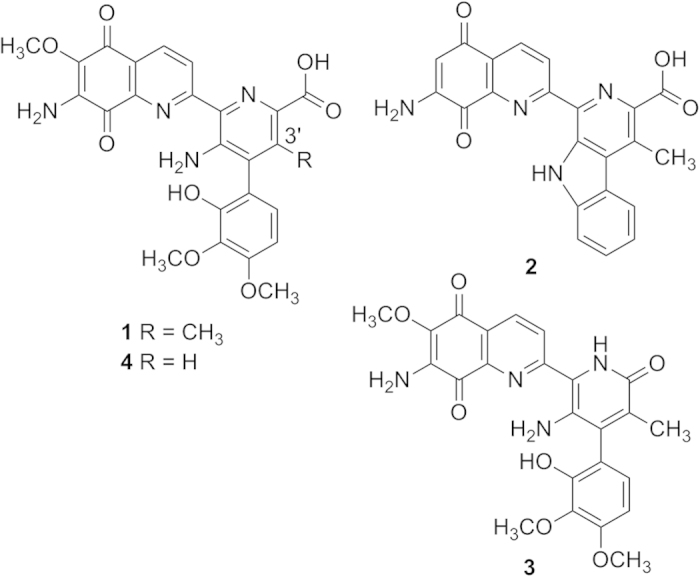
Chemical structures of streptonigrin (STN, 1), lavendamycin (2), streptonigron (3), and 3′-desmethylstreptonigrin (4).

**Figure 2:**
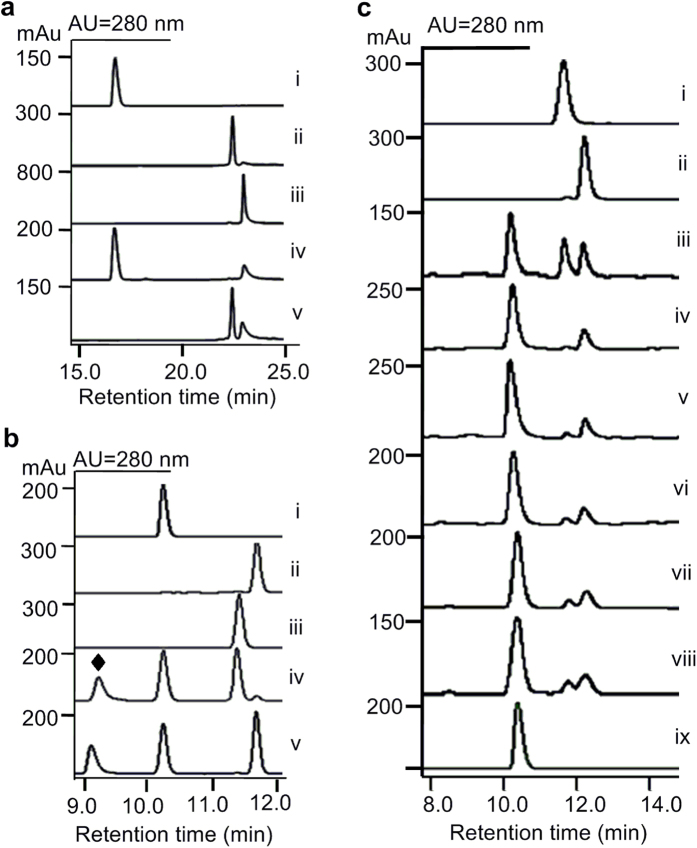
HPLC profiles of biochemical assays of StnR, StnQ1, and StnK3. (**a**) (i) l-Trp; (ii) β-methyl indolepyruvate; (iii) indolepyruvate; (iv) StnR incubated with l-Trp and α-KG; (v) StnQ1 incubated with indolepyruvate and SAM. (**b**) (i) l-Trp; (ii) (2*S*,3*S*)-β-MeTrp; (iii) (2*S*,3*R*)-β-MeTrp; (iv) StnR/Q1 incubated with l-Trp and SAM; (v) StnR/K3/Q1 incubated with l-Trp and SAM. (**c**) (i) (2*S*,3*R*)-β-MeTrp; (ii) (2*S*,3*S*)-β-MeTrp; (iii) StnR (20 μM) incubated with racemic β-methyl indolepyruvate and l-Trp; (iv-viii) StnR (2 μM) incubated with racemic β-methyl indolepyruvate and l-Trp for 0.5 h (iv), 1.0 h (v), 2.0 h (vi), 4.0 h (vii), 8.0 h (viii); (ix) l-Trp. ♦ *S*-adenosylhomocysteine (SAH) confirmed by MS.

**Figure 3:**
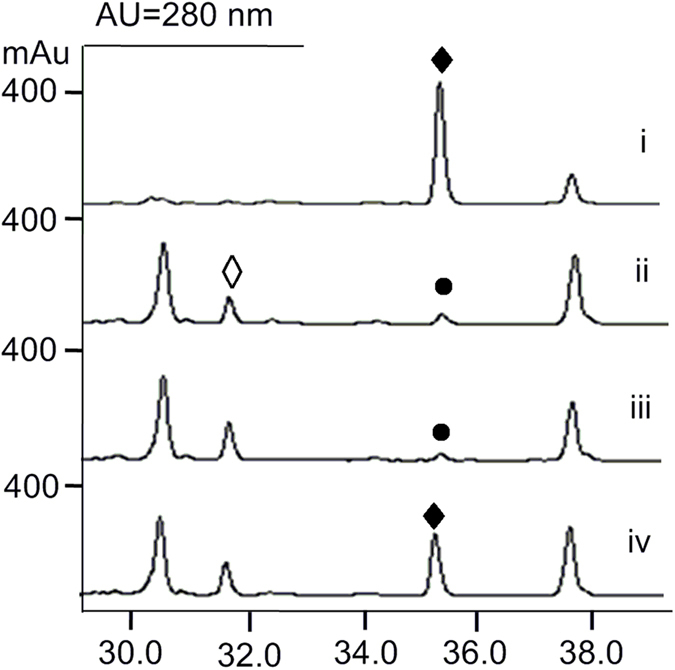
HPLC profiles of fermentation extracts. (i) STN-producing strain wild-type; (ii) Δ*stnQ1* mutant; (iii) Δ*stnQ1* mutant fed with (2*S*,3*R*)-β-MeTrp; (iv) Δ*stnQ1* mutant fed with (2*S*,3*S*)-β-MeTrp. ♦ **1** and ◊ **4**; • is not STN confirmed by MS analysis.

The *in vitro* characterization of StnR/K3/Q1 showed that both (2*S*,3*R*)-β-MeTrp and (2*S*,3*S*)-β-MeTrp can be synthesized by the STN biosynthetic pathway, but the production of the (3*S*)-epimer is favored by action of the epimerase StnK3. Taken together with the (2*S*,3*S*)-selectivity of StnR, (2*S*,3*S*)-β-MeTrp seems to be the predominant intermediate of the STN biosynthetic pathway on a biochemical level. However, this finding seemingly contradicts previous studies in which (2*S*,3*R*)-β-MeTrp was obtained by incubating a cell-free extract of the STN producer *S. flocculus* with tryptophan and SAM^31^,^32^. To determine the actual biosynthetic intermediate of the STN assembly, the inactivation mutant Δ*stnQ1* was constructed by replacement of *stnQ1* with an *aac(3)IV* gene cassette (Materials and Methods, Figure S3). When this mutant was fermented and the resulting broth was monitored by high performance liquid chromatography (HPLC, Fig. 3), the production of **1** was completely abolished and a new compound (**4**) was accumulated (Fig. 3, trace ii). Mass spectral (MS) analysis gave quasimolecular ion peaks [M + H]^+^ of 493.2 and [M + Na]^+^ of 515.1. The loss of 14 mass units in comparison to **1** indicates that **4** might be a desmethyl streptonigrin analogue. Subsequently, about 2 mg of **4** was purified from 14 L fermentation broth (Materials and Methods). High-resolution electrospray ionization (ESI)-MS analysis revealed the molecular formula C_24_H_20_N_4_O_8_ (calcd. 493.1354, found 493.1351 [M + H]^+^). The ^1^H NMR spectrum of **4** lacks the signal for a methyl group that occurs at δ_H_ 2.12 ppm (s, 3H) in other STN analogues^33^, and shows a singlet aromatic proton signal occurring at δ_H_ 7.68 ppm (s, 1H) instead (Table S3 and Figure S4). The ^13^C NMR spectrum shows 24 carbon signals. Among them, there are three signals belonging to methoxyl groups (δ_C_ 59.7, 55.7, 60.2 ppm) while all other signals represent aromatic or carbonyl carbon atoms. (Table S3 and Figure S5). Without any apparent *C*-methyl group signal visible, MS and comprehensive NMR analyses (Figures S6 and S7) confirmed the identity of **4** as 3′-desmethylstreptonigrin (Fig. 1). Moreover, the accumulation of **4** by the Δ*stnQ1* mutant indicates that the enzymes involved in the follow-up pathway of STN biosynthesis can tolerate the slight change at the 3′-position, and effectively incorporate l-Trp into the pathway.

### Verification of the intermediate of STN

With both isomers of (2*S*)-β-MeTrp and the Δ*stnQ1* mutant available, feeding experiments were carried out to confirm the biochemical data. When (2*S*,3*R*)-β-MeTrp was fed to the Δ*stnQ1* mutant, the STN production remained abolished (Fig. 3, trace iii). However, the STN production was restored by feeding of (2*S*,3*S*)-β-MeTrp to the Δ*stnQ1* mutant, pointing towards (2*S*,3*S*)-β-MeTrp as the biosynthetic precursor of STN.

Even with this secondary evidence in hand, it cannot be ruled out that (2*S*,3*R*)-β-MeTrp is the biosynthetic precursor of (2*S*,3*S*)-β-MeTrp and that (2*S*,3*R*)-β-MeTrp is converted to (2*S*,3*S*)-β-MeTrp by StnK3. If the replacement of *stnQ1* with the *aac(3)IV* gene cassette caused a polar effect leading to inactivation of StnK3, the conversion of (2*S*,3*R*)-β-MeTrp to (2*S*,3*S*)-β-MeTrp would then be disrupted. Genetic complementation of *stnQ1* is hard to perform, because *S. flocculus* CGMCC 4.1223 is resistant to most antibiotics used in genetic manipulation experiments. Instead, a biochemical experiment was carried out to evaluate the potential conversion of (2*S*,3*R*)-β-MeTrp to (2*S*,3*S*)-β-MeTrp by StnK3. When StnK3 was incubated with (2*S*,3*R*)-β-MeTrp for 2 hours at double concentration of StnK3, the lack of conversion (data not shown) affirmed that (2*S*,3*R*)-β-MeTrp is not a substrate of StnK3. Moreover, these data implicate that (2*S*,3*R*)-β-MeTrp is not the precursor of (2*S*,3*S*)-β-MeTrp in the biosynthetic pathway of STN. The identification of (2*S*,3*R*)-β-MeTrp in fermentation cultures of the streptonigrin producers is in line with this assumption and designates (2*S*,3*R*)-β-MeTrp as a pathway shunt-product while (2*S*,3*S*)-β-MeTrp is confirmed as the real biosynthetic precursor of streptonigrin^28^,^29^. Furthermore, (2*S*,3*R*)-β-MeTrp production by dialyzed cell-free extracts of the *S. flocculus*^31^ can be explained taking into account the small size of epimerase StnK3 (129 aa). As smaller proteins are easily lost during the dialysis process, the controlled production of (2*S*,3*S*)-β-MeTrp was lost as a result.

## Conclusion

The formations of (2*S*,3*R*)-β-MeTrp by StnR and StnQ1 and of (2*S*,3*S*)-β-MeTrp by StnR, StnQ1, and StnK3 were characterized *in vitro*. It has been shown that the aminotransferase StnR is not only specific for l-tryptophan as an amino donor, but also preferentially catalyzes the transamination of the amino acceptor (*S*)-β-methyl indolepyruvate over the epimer (*R*)-β-methyl indolepyruvate. STN production was abolished in the knockout mutant Δ*stnQ1* and could be restored only by feeding of (2*S*,3*S*)-β-MeTrp, but not of (2*S*,3*R*)-β-MeTrp. Along with the accumulation of (2*S*,3*R*)-β-MeTrp in the *S. flocculus* wild type, (2*S*,3*R*)-β-MeTrp is established as a shunt-product while (2*S*,3*S*)-β-MeTrp is the true intermediate of the STN biosynthetic pathway.

**Figure 4:**
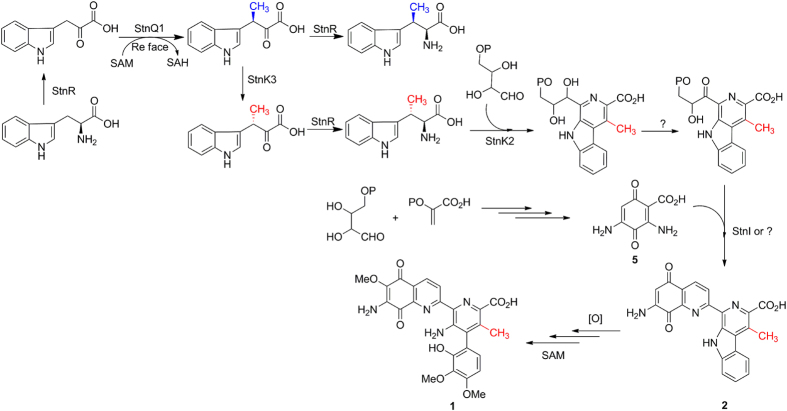
StnR/K3/Q1-catalyzed biosynthesis of (2*S*,3*R*)- and (2*S*,3*S*)-β-MeTrp from L-Trp and the modified biosynthetic pathway of STN.

Based on the current biochemical and genetic data, the following modified biosynthetic pathway of STN is proposed (Fig. 4). The sole aminotransferase StnR, that has been previously implicated in the biosynthesis of the aminoquinone moiety (**5**)^33^, was biochemically confirmed to catalyze the transaminations for the possible formation of (2*S*,3*R*)-β-MeTrp and (2*S*,3*S*)-β-MeTrp in this study. However, the epimerase StnK3 controls the specific formation of (2*S*,3*S*)-β-MeTrp in the biosynthetic pathway of STN. Eventually, (2*S*,3*S*)-β-MeTrp is stereospecifically recognized by StnK2 and incorporated into lavendamycin (**2**) which is modified in sequential oxidations and methylations to furnish **1**. In summary, this study sets the stage for a complete understanding of the biosynthetic mechanism of STN and for producing new streptonigrinoids by manipulation of the biosynthetic pathway.

## Materials and Methods

### General

The bacterial strains and plasmids used in this study are listed in Table S1. All *E. coli* strains were grown in liquid Luria-Bertani (LB) medium at 37 °C or 30 °C and 220 rpm. When used, antibiotics were supplemented at the following concentrations: apramycin (Apr, 50 μg mL^−1^), kanamycin (Kan, 50 μg mL^−1^), ampicillin (Amp, 100 μg mL^−1^), chloramphenicol (Cml, 25 μg mL^−1^), thiostrepton (Tsr, 30 μg mL^−1^). PCR amplifications were performed on a Veriti thermal cycler (Applied Biosystems, Carslbad, CA) using Taq DNA polymerase or KOD-plus high fidelity polymerase, which were purchased from Takara Co. Ltd Company (Dalian, China). Restriction enzymes and DNA ligase were purchased from Fermentas (Thermo Fisher Scientific Inc, MA, USA) or NEB companies (Gene, England). Gel extraction kits were purchased from Omega Bio-tek (GA, USA). Unless otherwise stated, other biochemicals and chemicals were purchased from standard commercial sources. All DNA manipulations in *E. coli* and *Streptomyces* were performed according to standard procedures. DNA sequencing was performed by Jie Li Biotech Co. Ltd. (Shanghai, China). All primers used in this work were synthesized by Sangong Biotech Co. Ltd. Company (Shanghai, China) and are listed in Table S2.

### Cloning, expression, and purification of StnQ1, StnR, and StnK3

PCR-amplified products of *stnQ1*, *stnK3*, and *stnR* were digested using *Nde*I-*Xho*I. The resulting fragments were cloned into pET28a digested with the same restriction enzymes to yield the expression plasmids pLS2002-*stnQ1*, pLS2003-*stnK3*, and pLS2004-*stnR*. *E. coli* BL21(DE3) cells harboring the expression plasmids were cultured at 37 °C and 220 rpm in LB medium supplemented with 50 μg mL^−1^ kanamycin (final concentration) to OD_600_ between 0.4 and 0.6. The cultures were then incubated on ice for 10 min before addition of 0.4 mM (final concentration) IPTG to induce protein expression. The cells were further cultured at 16 °C for 20–24 h. After harvesting by centrifugation (3,500 rpm, 15 min, 4 °C), the cells were re-suspended in 40 mL buffer A (50 mM Tris-HCl, pH 8.0, 0.5 M NaCl, and 10% glycerol) and lysed by sonication on ice for 40 min. Cellular debris was removed by centrifugation (12,500 rpm, 45 min, 4 °C), and the supernatant was loaded onto nickel-affinity chromatography columns. The proteins were eluted by an increasing gradient of buffer B (500 mM imidazole in buffer A). Purified proteins were concentrated and exchanged into buffer C (50 mM Tris-HCl, pH 8.0, 50 mM NaCl, and 5% glycerol) with the centriprep filters (Amicon). The protein purity was >90% judged by SDS-PAGE analysis and proteins were stored in buffer C at −80 °C. Protein concentrations were determined by Bradford assay using bovine serum albumin as a standard.

### Biochemical assays of StnR, StnQ1, StnK3

(1). Assays for the StnR activity with l-Trp were performed in a 100 μL solution at 30 °C for 1 h in the presence of 1 mM l-Trp, 1 mM α-ketoglutaric acid (α-KG) and 5 μM StnR in 50 mM 4-(2-hydroxyethyl)-1-piperazine-ethanesulfonic acid (HEPES, pH 8.0) buffer.

(2). Assays for the StnQ1 activity with indolepyruvate (InPy) as the substrate were performed in a 100 μL solution at 30 °C for 1 h. The assay mixtures contained 50 mM HEPES buffer (pH 8.0), 1 mM InPy, 1 mM S-adenosylmethionine (SAM) and 5 μM StnQ1.

(3). Assays for the StnQ1/R or StnQ1/R/K3-catalyzed coupled reaction with l-Trp as the substrate were performed in a 100 μL solution at 30 °C for 1 h in the presence of 1 mM l-Trp, 1 mM SAM and 5 μM StnQ1, 5 μM StnR, and 5 μM StnK3 in 50 mM HEPES buffer (pH 8.0).

(4). Assays for the StnK3 reaction with (2*S*,3*R*)-β-MeTrp as a surrogate were performed in a 100 μL solution at 30 °C for 2 hours in the presence of 1 mM (2*S*,3*R*)-β-MeTrp, and 10 μM StnK3 in 50 mM HEPES buffer (pH 8.0).

(5). Assays for the StnR activity with racemic β-methyl indolepyruvate were performed in a 100 μL solution at 30 °C for 1 hour in the presence of 1 mM β-methyl InPy, 1 mM l-Trp, and 20 μM StnR or 2 μM StnR in 50 mM HEPES buffer (pH 8.0).

All of the reactions were quenched by addition of 200 μL methanol to precipitate proteins and solvent was removed by speed-vac. The resulting residues were re-dissolved in 80 μL methanol, and analyzed with HPLC or LC-HRMS in positive mode. The mobile phase was comprised of solvent A (Milli-Q water) and solvent B (acetonitrile). HPLC analyses were performed on an Agilent HPLC Series 1200 and LC-HRMS analyses were performed on an Agilent 1200 series coupled with a 6530 Accurate-Mass Q-TOF MASS Spectrometer using a ZORBAX SB-C18 (Agilent, 5 μm, 150 × 4.6 mm) column under the following conditions: 5% to 40% B (0–15 min); 40–100% B (15–20 min); 100% B (20–25 min); 100% to 5% B (25–26 min); 5% B (26–30 min) at a flow rate of 0.6 mL min^−1^ (0.5 mL min^−1^ for LC-MS and LC-HR-MS) and UV detection at 280 nm. When detecting β-methyl indolepyruvate acid, solvent A contained 1‰ formic acid and the flow rate was changed to 0.3 mL min^−1^.

### Construction of the Δ*stnQ1* mutant

The gene *stnQ1* was inactivated using a standard PCR targeting strategy based on the λ-Red recombination functions and the *aac(3)IV* + *ori*T cassette. The constructed plasmid pLS1100 was transformed into *E. coli* BW25113/pIJ790 and the cassette was introduced by electroporation. The positive clones with the target gene replaced by the cassette were identified by restriction endonuclease reaction.

The resulting gene inactivation construct pLS1101 was then transformed into *E. coli* ET12567/pUZ8002 to generate *E. coli* ET12567/pUZ8002/pLS1101 and the conjugation was performed using spores of *S. flocculus* CGMCC 4.1223 which were treated according to standard methods. After *E. coli* ET12567/pUZ8002/pLS1101 growth in LB medium to an OD_600_ of 0.4–0.6, the cells were washed twice and re-suspended in LB medium. Spores of *S. flocculus* CGMCC 4.1223 were produced in SFM medium (soybean meal 2% in tap water was sterilized and filtered. The filtrate was mixed with 2% mannitol and 2% agar for sterilization again). The donor and recipient cells were mixed and spread on IWL-4 (soluble starch 1%, tryptone 0.2%, yeast extract 0.1%, K_2_HPO_4_ 0.1%, MgSO_4_ · 7 H_2_O 0.1%, NaCl 0.1%, (NH_4_)_2_SO_4_ 0.2%, CaCO_3_ 0.2%, FeSO_4_ · 7 H_2_O 1 mg L^−1^, MnCl_2_ 1 mg L^−1^, ZnSO_4_·7H_2_O 1 mg L^−1^, pH 7.0–7.4) and grown for 16–20 h. The exconjugants were selected with 35 μg mL^−1^ apramycin, 25 μg mL^−1^ thiostrepton, and 50 μg mL^−1^ trimethoprim after cultivation for 4 to 6 days. Double-crossover mutants were preselected by the phenotype showing Kan^S^/Apr^R^ and subsequently identified through diagnostic PCR with corresponding primers (Table S2 and Figure S3).

### Fermentation and analysis of *S. flocculus* CGMCC 4.1223 wild type and Δ*stnQ1* mutant strains

Wild type and mutant strains were fermented under the same culture conditions. The strains were firstly cultured in seed medium TSBY (TSB 3% and yeast extract 0.5%) for 2–3 days, and then 1% inocula were cultured in production medium (glucose 2.5%, soybean 1.5%, NaCl 0.5%, KCl 0.05%, MgSO_4_ · 7 H_2_O 0.025%, K_2_HPO_4_ 0.3%, Na_2_HPO_4_ · 12 H_2_O 0.3%) for 7–8 days. After finishing the fermentation, the broths were harvested by centrifugation and extracted with ethyl acetate. The resulting organic phase was concentrated and analyzed by HPLC with UV detection at 210, 245, and 375 nm under the following conditions: 10% to 70% B (linear gradient, 0–40 min), 70% to 100% B (linear gradient, 41–45 min), 100% B (45–50 min) at a flow rate of 0.6 mL min^−1^. A is Milli-Q water and B is acetonitrile.

### Feeding of Δ*stnQ1* mutant strains

After the Δ*stnQ1* mutant strains were fermented for two days, (2*S*,3*R*)- or (2*S*,3*S*)-β-methyltryptophan were added to a final concentration of 5 mM, respectively. After finishing the fermentation on the 7th day, the broths were harvested by centrifugation and extracted with ethyl acetate and analyzed by HPLC as described previously.

### Purification and Characterization of 3′-desmethylstreptonigrin (4)

In order to purify compound **4**, about 14 L fermentation broths were adjusted to pH 9.5 with 10 M NaOH solution and extracted with ethyl acetate (12 L × 2). The aqueous phase was collected, adjusted to pH 5 with 12 M HCl, and extracted with ethyl acetate (15 L × 2). After evaporation of the combined organic phase, the crude extract was subjected to reverse phase column chromatography (C_18_, 50 μm, YMC, Japan) that was eluted with H_2_O/CH_3_OH (60:40-30:70). The fractions containing **4** judged by HPLC analysis were collected and concentrated. The resultant residues were re-dissolved in CH_3_OH, loaded on Sephadex LH-20 column, and eluted with CH_3_OH. The fractions containing the pure **4** were collected and dried. Finally, 2.0 mg of **4** was obtained and subjected to NMR analysis that was carried out on a Bruker AV-500MHz NMR spectrometer with tetramethylsilane (TMS, 0.0 ppm) as the internal standard. Its molecular formula was established as C_24_H_20_N_4_O_8_ based on the HR-ESI-MS (*m/z* 493.1351 [M + H]^+^). The ^1^H and ^13^C NMR data were summarized in Table S3 and Figures S4-7.

## Additional Information

**How to cite this article**: Kong, D. *et al*. Identification of (2*S*,3*S*)-β-Methyltryptophan as the Real Biosynthetic Intermediate of Antitumor Agent Streptonigrin. *Sci. Rep*. **6**, 20273; doi: 10.1038/srep20273 (2016).

## Supplementary Material

Supplementary Information
