# Supplementary material for: Identification of (2S,3S)-β-Methyltryptophan as the Real Biosynthetic Intermediate of Antitumor Agent Streptonigrin
Source: Sci Rep. 2016 Feb 5;6:20273. doi: 10.1038/srep20273 (PMC4742848; doi:10.1038/srep20273)

Supplementary Information

Identification of (2*S*,3*S*)-β-Methyltryptophan as the Real Biosynthetic Intermediate of Antitumor Agent Streptonigrin

Dekun Kong[1], Yi Zou[1], Zhang Zhang[2], Fei Xu[1], Nelson L. Brock[1], Liping Zhang[2], Zixin Deng[1] and Shuangjun Lin[1*]

[1]State Key Laboratory of Microbial Metabolism, Joint International Laboratory on Metabolic & Developmental Sciences, School of Life Sciences & Biotechnology, Shanghai Jiao Tong University, 800 Dongchuan Road, Shanghai 200240, P. R. China

[2]College of Life Science, Hebei University, Baoding 071002, P. R. China

Correspondence and requests for materials should be addressed to S.-J.L. (email: linsj@sjtu.edu.cn)

1. Tables

1.1 Table S1. Strains and plasmids used in this study

Strains/plasmids	Relevant phenotype	Source/Ref
**Strains**		
E. coli DH10B	F-mcrA Δ(mrr-hsdRMS-mcrBC)φ80dlacZ ΔM15 ΔlacX74 deoR recA1 endA1 ara Δ139 D (ara, leu)1697 galUgalK λ-rspLnupG	GIBCO BRL
E. coli ET12567/pUZ8002	*rec*F, *dam-*, *dcm-*, *hsd*S, Cml^R, Kan^R	1
E. coli BW25113	Δ(araD-araB)567, ΔlacZ4787(::rrnB-4), lacIp-4000(lacIQ), λ-, rpoS369(Am), rph-1, Δ(rhaD-rhaB)568, hsdR514	2
E. coli BL21(DE3)pLysS	F-ompThsdSB(rB-mB-) gal dcm (DE3) pLysS (Chl^R)	Novagen
*S. flocculus*CGMCC 4.1223	Wild type producer of Streptonigrin	CGMCC
LS1101(*ΔStnQ1*)	*StnQ1* inactivation mutant of CGMCC 4.1223	This study
LS1102	*E. coli* BL21(DE3)harbors the expression plasmid pLS1102-*StnQ1*	This study
LS1103	*E. coli* BL21(DE3)harbors the expression plasmid pLS1103-*StnK3*	This study
LS1104	*E. coli* BL21(DE3)harbors the expression plasmid pLS1104-*StnR*	This study
**Plasmids**		
$pBSK^+$	*bla, lacZ*, pUC origin, general clone vector	Stragagene
pET28a	Kan^R , pBR322 origin, T7 promoter, general protein expression vector	Novagen
pIJ790	Chl^R, including λ-RED (*gam, bet, exo*) for PCR-targeting	3
pIJ773	Apr^R, source of *acc(3)IV* and *oriT* fragment	3
pJTU1289	*oriT* region of pJTU1278 was deleted for PCR-targeting	4
pLS1100	The cosmid 9A9 was digested with *BamHI*, and then the 15.2 kb DNA fragment was ligated with pJTU1289 to generate pLS1100, which was used for Δ*stnQ1* mutant	This study
pLS1101	The *StnQ1* in pLS1100 was substituted by*aac(3)IV*+*oriT* cassette	This study
pLS1102-*stnQ1*	The *stnQ1* PCR product (*Nde*I-*Xho*I digested) was ligated into the same sites of pET28a for *stnQ1* expression	This study
pLS1103-*stnK3*	The *stnK3* PCR product (*Nde*I-*Xho*I digested) was ligated into the same sites of pET28a for *stnK3* expression	This study
pLS1104-*stnR*	The*stnR* PCR product (*Nde*I-*Xho*I digested) was ligated into the same sites of pET28a for *stnR* expression	This study

1.2 Table S2. Primers used in this study

Primers code	Sequences of primers (5'-3')
stnQ1TarF	GGGGGTCTTCGAGCTGTTGCGGGACGAGAAATCCCTCGAATTCCGGGGATCCGTCGACC
stnQ1TarR	CGTCACCTTCCTCGTTCCGCGGCCTGATCGTGGTGTCGGTGTAGGCTGGAGCTGCTTC
stnQ1VtarF	CCCGGACACCGCGTTCAACGG
stnQ1VtarR	GGTAGGTGTGCGGGGCGCCG
stnQ128aF	CCTTA***CATATG***GTGTCGCCGCTGAACACG (*Nde*I site underlined)
stnQ128aR	CCTTA***CTCGAG***TCACGACGCCTCCAG (*XhoI* site underlined)
stnK3 28aF	CCTTA***CATATG***ATGGGCAGCAGCCATC(*Nde*I site underlined)
stnk3 28aR	CCTTA***CTCGAG***TCACAGGCGGTCCCCC(*XhoI*site underlined)
stnR 28aF	CCTTA***CATATG***ACCGCTCCGGTCCGCC(*Nde*I site underlined)
stnR 28aR	CCTTA***CTCGAG***TCAGCGCGAGCCGGCGG(*XhoI* site underlined)

Table S3. NMR data of the compound 4

Position	^{1}H NMR	^{13}C NMR
2		159.99
3	9.07 (d, J=8.5 Hz, 1H)	126.25
4	8.40 (d, J=8.5 Hz, 1H)	133.42
4a		126.87
5		175.85
6		135.77
7		141.52
8		180.21
8a		144.06
1′-CO		165.87
2′		134.08
3′	7.68 (s, 1H)	128.11
4′		133.47
5′		146.19
6′		132.02
7′		116.51
8′		148.02
9′		136.63
10′		153.27
11′	6.68 (d, J=8.5 Hz, 1H)	104.10
12′	6.93 (d, J=8.5 Hz, 1H)	124.76
6-OCH_3	3.83 (s, 3H)	59.67
9′-OCH_3	3.85 (s, 3H)	55.72
10′-OCH_3	3.77 (s, 3H)	60.24

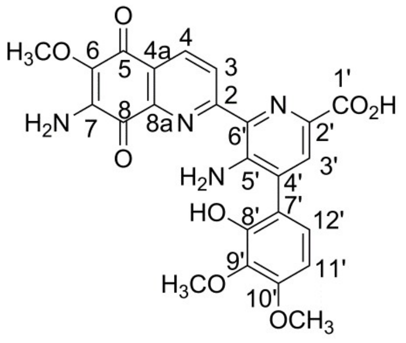

4

2. Figures

2.1 Figure S1. Allignment of StnQ1/K3/R and MarI/H/G

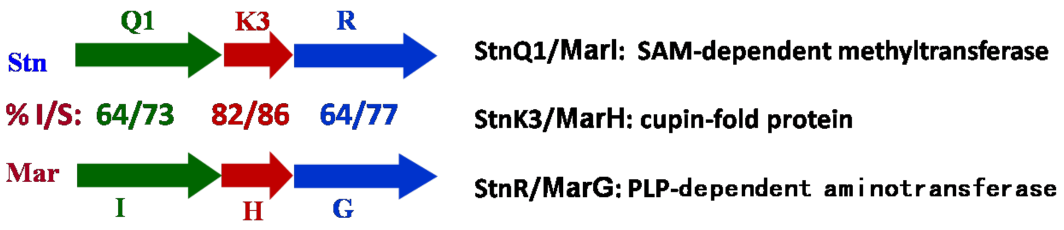

Figure S1. Identity (I) and similarity (S) of StnQ1/K3/R to MarI/H/G

2.2 Figure S2. Expression of StnQ1, StnK3, and StnR

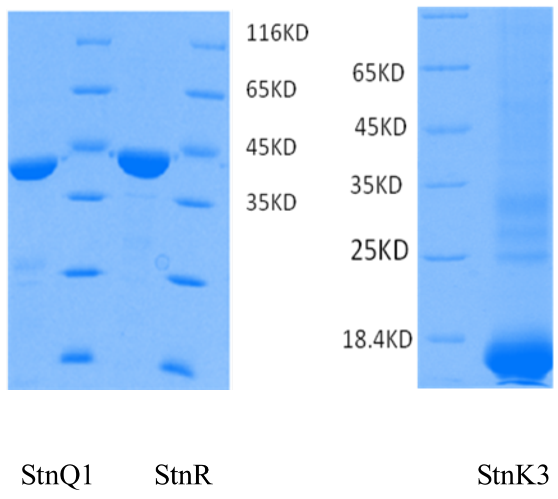

Figure S2. SDS-PAGE of the protein StnQ1, StnR, and Stnk3

2.3 Figure S3. Construction of the Δ*stnQ1* mutant

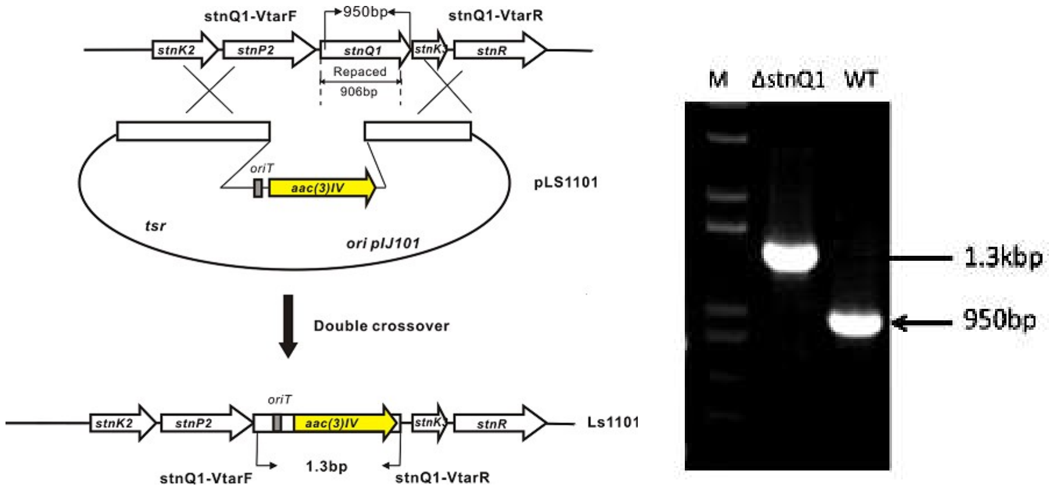

a b

Figure S3. Construction of the Δ*stnQ1* mutant. **a**. The scheme of *stnQ1* gene replacement with apramycin-resistant/oriT cassette (*aac(3)IV-oriT*); **b**. Confirmation of the inactivation of *stnQ1* by insertion of apramycin-resistant/oriT cassette (*aac(3)IV-oriT*) by PCR using the primers listed in Table S2

2.4 Figure S4. ^{1}H NMR spectrum of 4 (500 MHz, DMSO-d$_6$)

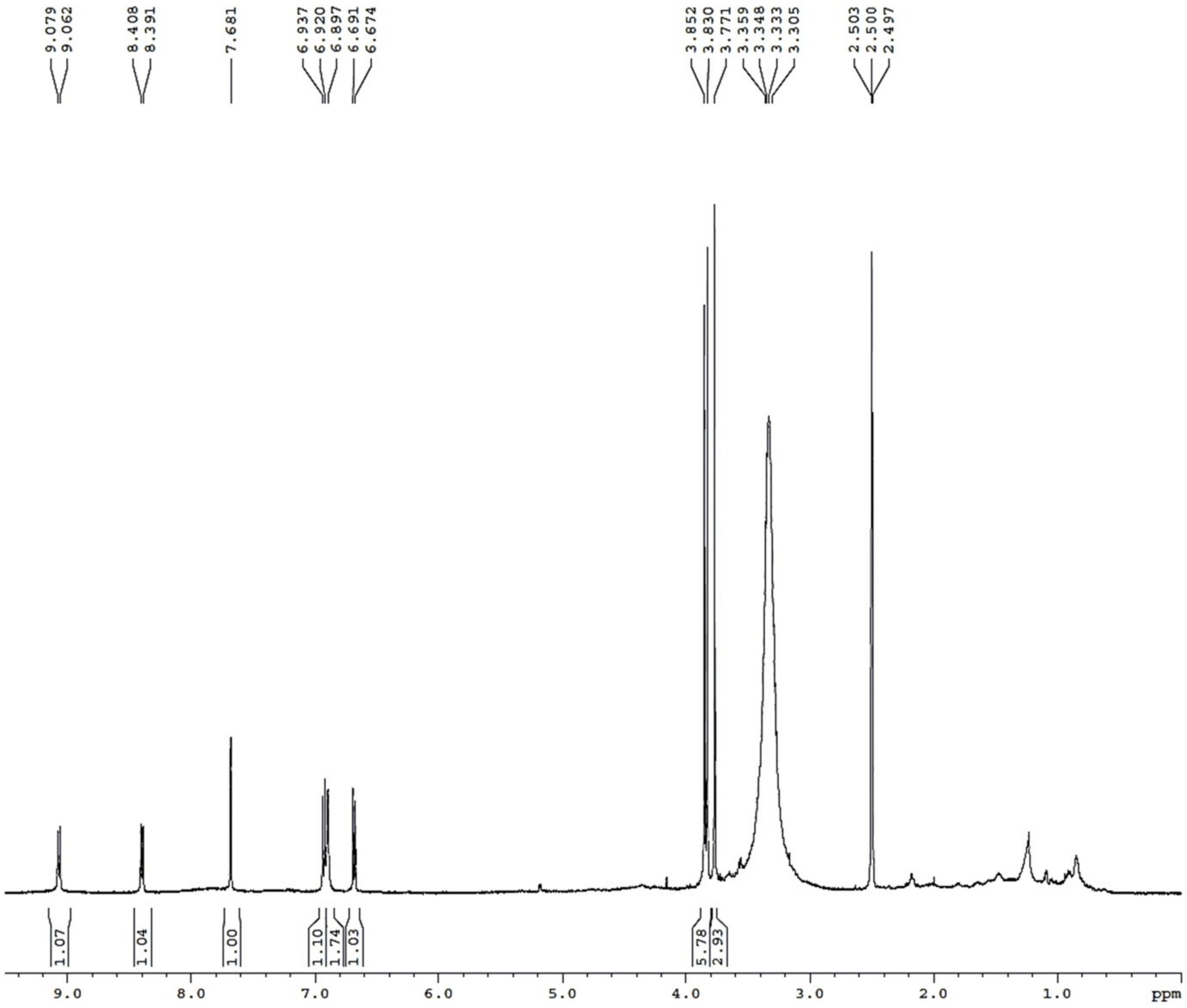

2.5 Figure S5. ^{13}C NMR spectrum of 4 (125 MHz, DMSO-d_6)

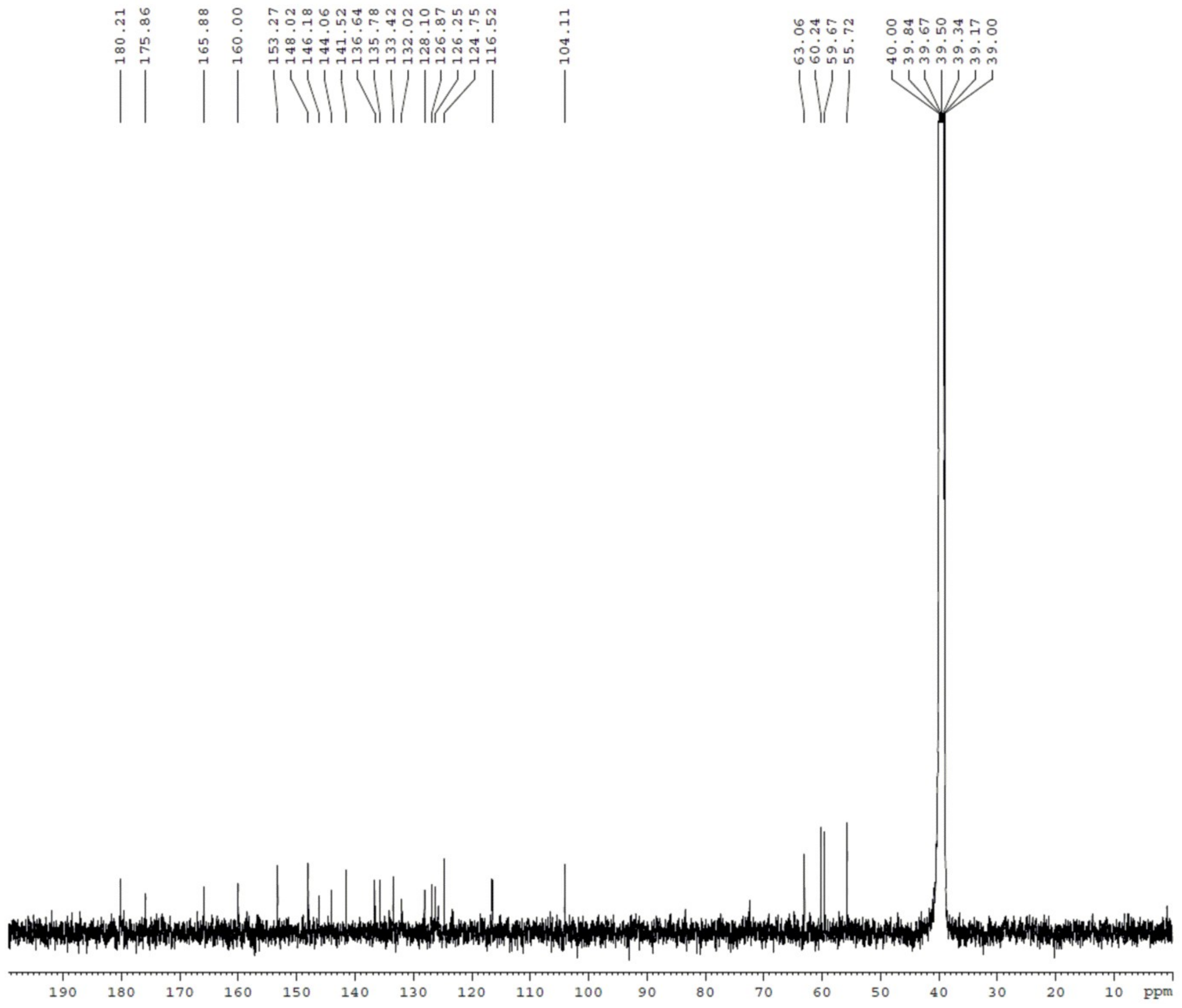

2.6 Figure S6. HMQC NMR spectrum of 4 (DMSO-d_6)

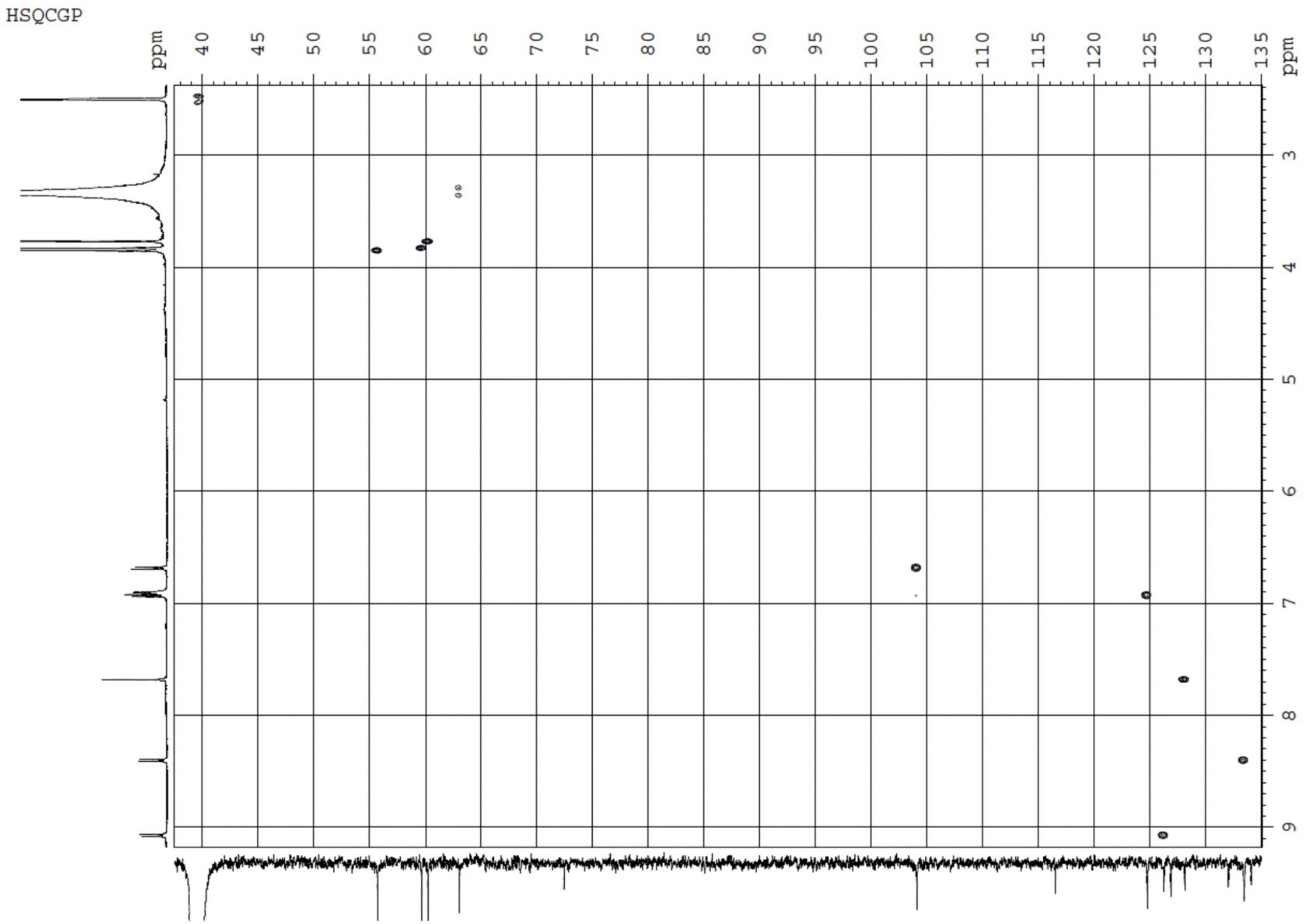

2.7 Figure S7. HMBC NMR spectrum of 4 (DMSO-d_6)

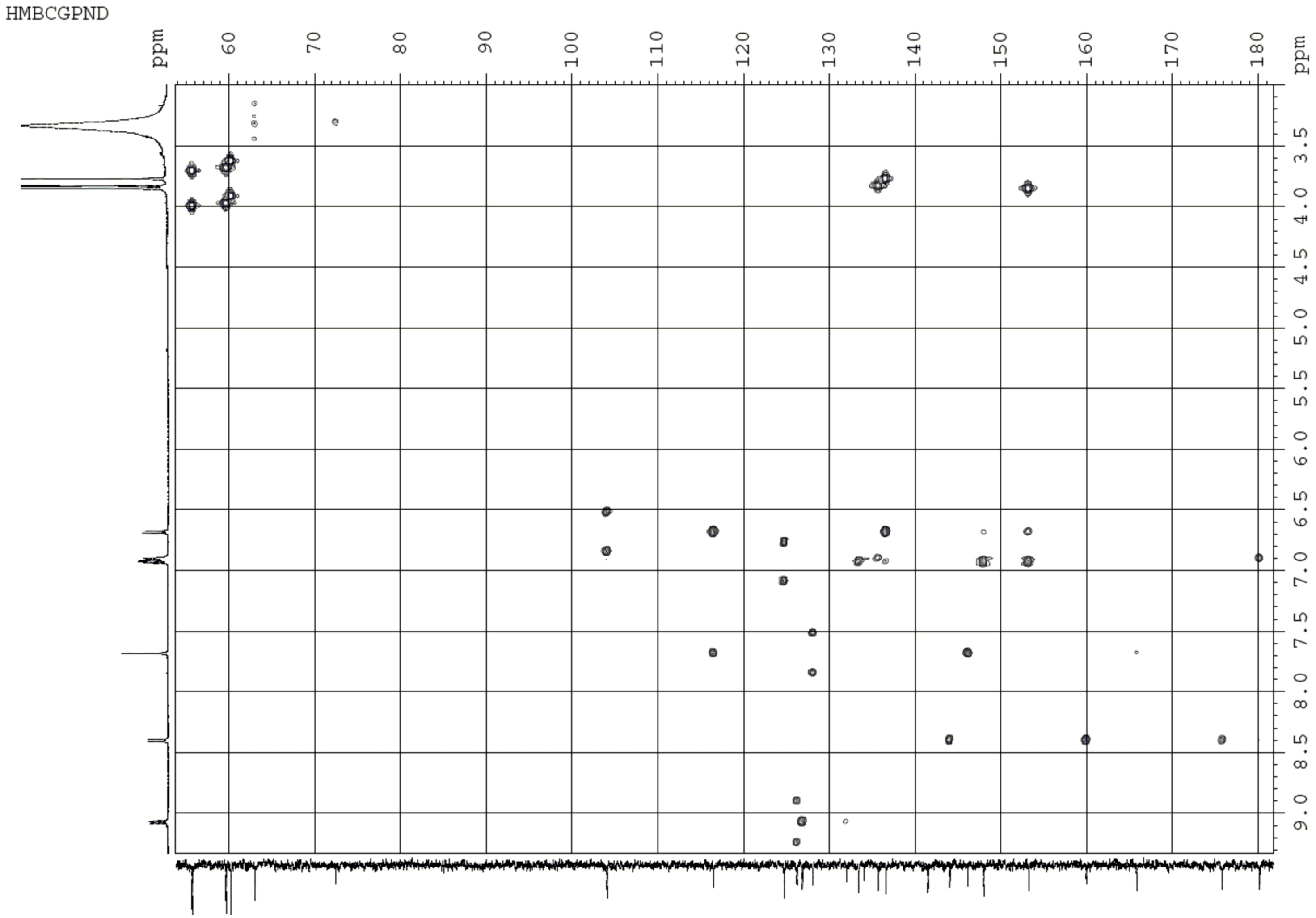

Supplement: Supplementary Information [file srep20273-s1.pdf]
